# Protective Effects of Naringenin from *Citrus sinensis* (var. Valencia) Peels against CCl_4_-Induced Hepatic and Renal Injuries in Rats Assessed by Metabolomics, Histological and Biochemical Analyses

**DOI:** 10.3390/nu14040841

**Published:** 2022-02-17

**Authors:** Naglaa M. Ammar, Heba A. Hassan, Heba M. I. Abdallah, Sherif M. Afifi, Abdelbaset M. Elgamal, Abdel Razik H. Farrag, Abd El-Nasser G. El-Gendy, Mohamed A. Farag, Abdelsamed I. Elshamy

**Affiliations:** 1 Therapeutic Chemistry Department, Pharmaceutical and Drugs Research Institute, National Research Centre, Giza 12622, Egypt; nm.ammar@nrc.sci.eg (N.M.A.); ha.el-saud@nrc.sci.eg (H.A.H.); 2 Pharmacology Department, Medical Research and Clinical Studies Institute, National Research Centre, Giza 12622, Egypt; heba21_5@yahoo.com; 3 Pharmacognosy Department, Faculty of Pharmacy, University of Sadat City, Sadat City 32897, Egypt; shshsh38@hotmail.com; 4 Chemistry of Microbial and Natural Products Department, Pharmaceutical and Drugs Research Institute, National Research Centre, Giza 12622, Egypt; algamalgene@yahoo.com; 5 Department of Pathology, Medical Research and Clinical Studies Institute, National Research Centre, Giza 12622, Egypt; ar.hussein@nrc.sci.eg; 6 Medicinal and Aromatic Plants Research Department, Pharmaceutical and Drugs Research Institute, National Research Centre, Cairo 12622, Egypt; aggundy_5@yahoo.com; 7 Pharmacognosy Department, College of Pharmacy, Cairo University, Cairo 11562, Egypt; mfarag73@yahoo.com; 8 School of Forestry and Biotechnology, Zhejiang A&F University, Hangzhou 311300, China; 9 Chemistry of Natural Compounds Department, Pharmaceutical and Drugs Research Institute, National Research Centre, Giza 12622, Egypt

**Keywords:** *Citrus sinensis* (var. Valencia), naringenin, CCl_4_ hepatic and renal injury, metabolomics, biochemical markers, histopathological examination

## Abstract

*Citrus* fruits are grown worldwide for their special nutritive and several health benefits. Among citrus bioactives, naringenin, a major flavanone, exhibits a potential hepatoprotective effect that is not fully elucidated. Herein, serum biochemical parameters and histopathological assays were used to estimate the hepatoprotective activity of naringenin, isolated from *Citrus sinensis* (var. Valencia) peels, in CCl_4_-induced injury in a rat model. Further, GC–MS-based untargeted metabolomics was used to characterize the potential metabolite biomarkers associated with its activity. Present results revealed that naringenin could ameliorate the increases in liver enzymes (ALT and AST) induced by CCl_4_ and attenuate the pathological changes in liver tissue. Naringenin decreased urea, creatinine and uric acid levels and improved the kidney tissue architecture, suggesting its role in treating renal disorders. In addition, naringenin increased the expression of the antiapoptoic cell marker, Bcl-2. Significant changes in serum metabolic profiling were noticed in the naringenin-treated group compared to the CCl_4_ group, exemplified by increases in palmitic acid, stearic acid, myristic acid and lauric acids and decrease levels of alanine, tryptophan, lactic acid, glucosamine and glucose in CCl_4_ model rats. The results suggested that naringenin’s potential hepato- and renoprotective effects could be related to its ability to regulate fatty acids (FAs), amino acids and energy metabolism, which may become effective targets for liver and kidney toxicity management. In conclusion, the current study presents new insights into the hepato- and renoprotective mechanisms of naringenin against CCl_4_-induced toxicity.

## 1. Introduction

The liver is one of the body’s vital organs, with critical functions in protein biosynthesis, glucose homeostasis, xenobiotics detoxification and nutrients utilization [1,2]. Liver disorders lead to two million deaths annually on a global scale [3]. Liver damage can be caused by alcohol intake, heavy metal intoxication, hepatitis virus infection, obstruction of the biliary tract and malnutrition [4]. Carbon tetrachloride (CCl_4_) continues to be one of the most commonly used toxins in the experimental studies on liver diseases. The liver is a substantial organ with unique metabolic functions [5] and is a primary target for CCl_4_ intoxication [6]. Liver injury is one of the main pathophysiological processes for developing hepatitis, liver fibrosis, cirrhosis and cancer, requiring the discovery of potential drug treatments [7]. In terms of therapeutic applications, although considerable innovations have been made in medicine, drugs used for the treatment of liver damage have many side effects and aggravate the disease [8]. Herbal medicines have recently sparked a lot of attention as alternative treatments, owing to their likely large safety margin due to being derived from dietary sources.

The edible fruits of the Citrus family, such as grapefruits, lemons, mandarins and oranges, are common fruits worldwide, with production yields of more than 90,000,000 tons [9]. More than 35% of citrus crops are used as the main backbone of the juice industry globally, including that of Egypt, as a major *Citrus* fruit producer [10]. Asides from their rich nutritional value, edible *Citrus* fruits are widely used in traditional medicine for the treatment of many ailments [9,11]. Several hydroxylated or methoxylated flavonoids have been reported from different *Citrus* peels, with potential biological activities, such as free radical scavenging and antiaromatase, antiestrogenic and anti-inflammation activities [9,12]. Naringenin is one of the main flavonoids of the different extracts of edible fruits and peels of the *Citrus* family [9]. It is a flavanone with significant biological and pharmaceutical effects, such as antitumor, anti-inflammatory antioxidant, antiatherogenic, antidepressant and antidiabetic effects [13]

Metabolomics analysis is a modern tool used in drug development, disease diagnosis and prognosis that offers a greater level of biochemical depth and knowledge than other systems biology omics techniques [14]. It can be used to measure perturbations in endogenous small molecules within the cells, tissues and biofluids of the body in response to a contaminant or other environmental change. Among metabolomics tools, gas chromatography–mass spectrometry (GC–MS) is the most popular metabolomics technology for quantitatively analyzing different metabolites in biological materials, separating and identifying vast pools of metabolites quickly and effectively [15]. 

Recently, scientists have focused on the biological and pharmaceutical applications of metabolites derived from edible plants and foods [16,17]. Some reports have shown that naringenin and its glycosides exert significant hepatoprotective effects [18,19,20]. However, the exact mechanismatic pathway of naringenin for liver protection in CCl_4-_induced liver damage is still unknown at the metabolite level. Understanding the mechanismatic pathways of herbal medicines is challenging, considering their chemical complexity [21], highlighting the need for tools that can provide a deeper understanding of naringenin’s action mechanisms in the treatment of liver diseases. It is also well-established that CCl_4_ induces renotoxicty in the form of multiple kidney injuries. Hence, the main goal of the current study was the investigation of the protective effects of naringenin against CCl_4_-induced hepato- and renotoxicity in rats based on metabolomics tools in combination with histopathological and biochemical parameter measurements.

## 2. Materials and Methods

### 2.1. Plant Materials, Extraction and Purification of Naringenin

The edible fruit of *Citrus sinensis* (var. Valencia) was collected in January 2019 by Prof. Dr. Mohamed A. Gibali, a senior botanist from El-Orman Botanic garden, Giza, Egypt (30°01′45″ N and 31°12′47″ E). The voucher sample (z11072-CSV1699) was deposited in the Herbarium garden. 

The peels of *C. sinensis* were isolated from the fruits, left to dry in the shade and then powdered. The dried and powdered peels (850 g) were macerated with 4 L of ethyl acetate for 3 days at room temperature, filtered and repeated 3 times. The overall extract was collected and dried under vacuum, affording 67 g of deep brownish gum. The extract was further fractionated via elution by CHCl_3_-MeOH (1:0, 4:1, 3:2, 2:3, 1:4 and 0:1) over sephadex column chromatography to afford 5 main fractions (CSV-1–CSV-5) after TLC analysis. Naringenin (472 mg) was afforded from fraction CSV-2 (2.43 g) via several purification attempts using Sephadex LH-20 CC columns eluted using mixtures of H_2_O-MeOH. The identification of naringenin was based on its NMR and MS data obtained using a Bruker 500 NMR spectrometer (USA) and JEOL JMS-700 instrument (Tokyo, Japan), respectively.

### 2.2. Chemicals and Drugs

Pyridine, methoxyamine hydrochloride, MSTFA (*n*-methyl-*n*-(trimethylsilyl) trifluoroacetamide), acetonitrile (99.7%), standard *n*-alkane (C8-C40), carbon tetrachloride or tetrachloromethane (CCl_4_) and trimethylchlorosilane were purchased from Sigma-Aldrich (St. Louis, MO, USA). All other used solvents and reagents were of analytical grade.

### 2.3. Pharmacological Study

#### 2.3.1. Animals

Thirty male Wistar rats (130–150 g b. w., age, 28–30 weeks) were obtained from the breeding unit of the National Research Centre (NRC) and were acclimatized for adapted under controlled standard environmental conditions (12 h dark and light cycle, temperature of 20–22 °C, 40–60% relative humidity) for several days. To reduce animal suffering, unnecessary disturbance was avoided, and the number of rats in each group was kept to a minimum. Squeezing, pressure and harsh maneuvering were avoided when treating the animals. The drug preparation and animal injection instruments were initially sterilized. Rats were anesthetized used whenever applicable. The doses of the naringenin were accurately calculated. Cadavers and tissues parts of the animals were handled with care with following the healthy hygiene principles, and dead bodies were incinerated in an NRC incineration system. The study was performed according to the ethics committee regulations of the NRC, which gave its approval according to the National Rules for Animal Welfare and Institutional Animal Ethical Committee (IAEC) (approval number: 20144). 

#### 2.3.2. Induction of Acute Liver Toxicity

The rats were randomly divided into five groups (six rats for each group). Group 1 (negative control) was given a single subcutaneous dose of olive oil (2.8 mL/kg, p.o.) followed by normal saline (0.5 mL/150 g, p.o.) every day for 3 consecutive days starting after 6 h from the olive oil administration. Group 2 (positive group) received a single subcutaneous dose of CCl_4_ (2.8 mL/kg, p.o., in olive oil, diluted by 1:1) following the previous described protocol [22], followed by administration of saline similarly to the control group. For groups 3–5 (treated groups), CCl_4_ was administered in a single subcutaneous dose (2.8 mL/kg) and they were treated with drugs starting at 6 h after CCl_4_ injection. Group 3 was given naringenin at a dose of 20 mg/kg. Group 4 was given naringenin at a dose of 40 mg/kg. Group 5 was given silymarin at a dose of 50 mg/kg. All drugs were orally administered at a volume of 0.5 mL/150 g daily for 3 days, and doses were selected according to previous reports [23,24,25]. Animals anesthesia occurred after three days of CCl_4_ injection. The collection of blood samples was performed from all animals in all groups and the samples were saved in sterilized tubes for the serum separation. The separation of sera was achieved via centrifugation for 15 min at 7000× *g* rpm. Then, the afforded serum samples were stored at −80 °C until further analyses, including the measurement of serum liver enzymes, kidney-related parameters and metabolite profiling via GC–MS. 

After obtaining serum samples, animals were sacrificed and the liver and kidneys were subsequently dissected for histopathological and immune–histochemical investigations.

#### 2.3.3. Biochemical Assays

The levels of serum enzymes in the liver, namely aspartate transaminase (AST) and alanine transaminase (ALT), as well as the kidney enzymes, creatinine, urea and uric acid, were assayed spectrophotometrically by using the available commercial reagent kits according to the procedures provided by the manufacturer (Biodiagnostic, Cairo, Egypt).

#### 2.3.4. Histopathologic Investigation

Aliquots of the tissues of the liver and kidneys were used and fixed for 1 week in formalin (10%) in phosphate-buffered normal saline. They were then washed for 2 h under running tap water and underwent dehydration in ethanol gradually, followed by embedding in paraffin wax. The sections were thereafter de-paraffinized with xylene and stained with hematoxylin and eosin. An examination was performed using an Olympus CX41 light microscope and SC100 digital camera, which was attached to a computer system.

#### 2.3.5. Immuno-Histochemical Assessment of Bcl-2 Expression in Kidneys and Liver

The expression of Bcl-2, an oncoprotein that causes apoptosis inhibition [26], was assessed, whereby the sections from the liver and kidneys were de-paraffinized and rehydrated. Next, a mixture of hydrogen peroxide (5%) in 100% methanol was left at room temperature for 10 min to block endogenous peroxidase activity. The sections were washed using phosphate-buffered saline (PBS) and then incubated with primary antibodies against Bcl-2. The levels of protein expression were measured using a streptavidin biotin peroxidase kit. The chromogen for Bcl-2 detection, diaminobenzidine (DAB), was used for tissues staining and then counterstained with hematoxylin [27]. For quantitation of BCl2 immuno-expression, six random non-overlapping microscopic fields from each sample per each group were analyzed, obtaining mean relative area percentages of immunohistochemical expression levels of Bcl2 in hepatic and renal tissue samples. All micrographs and data were obtained using a full-HD microscopic imaging system operated using the Leica application module for histological analysis.

#### 2.3.6. Preparation of Metabolomics Samples and GC–MS Analysis

Before sample processing, serum samples were melted on ice and metabolites were extracted and silylated using xylitol as an internal standard, as previous described [28]. Briefly, 100 μL of serum was combined with cold acetonitrile (100%, 200 μL) and centrifuged at 7000× *g* rpm for 15 min. Nitrogen gas was used to evaporate the supernatant until it was completely dry. A volume of 50 μL pyridine solution of methoxyamine (20 mg·mL^−1^) was first added to the dried residue, then incubated at 60 °C for 1 h to derivatize the metabolites. A second derivatization phase was performed by adding 100 μL MSTFA containing TMS (1%) to the mixture and incubating this for 1 h at 60 °C. The quality control (QC) sample was formed by mixing aliquots from all samples into a pooled sample that was used to analyze changes in MS response through measurement. Additionally, a hydrocarbon mixture standard (C8–C40) was analyzed. 

#### 2.3.7. GC–MS Analysis

Silylated products were separated following the procedure of Farag et al. [29] using an Agilent 5977B GC/MSD system equipped with a DB-5 column (30 m × 0.25 mm i.d. × 0.25 µm film thickness; Supelco) and coupled to a quadrupole mass spectrometer.

#### 2.3.8. Processing of GC–MS Data, Molecular Networking and Multivariate Data Analyses

The identification of metabolites in the sera was accomplished via comparisons of their retention indices (RI) relative to *n*-alkanes standards (C8–C40) alongside mass matching to NIST library database. Before mass spectral matching, AMDIS software (www.amdis.net accessed on 1 December 2021) was used to deconvolute the peaks. Data regarding metabolite abundance were retrieved using MS Dial software with default settings and Pareto scaling in preparation for multivariate data analysis. After this, the data were treated via principal component analysis (PCA), as well as orthogonal projection to latent structure discriminate analysis (OPLS-DA) via SIMCA-P software. The detection (LOD) and quantification (LOD) limits of 32 metabolites were also determined and signal-to-noise ratios of 3:1 and 10:1, respectively, were considered. 

#### 2.3.9. Statistical Analysis

The results of the biochemical analysis are presented as means ± standard error (SEM). The data were analyzed using one-way analysis of variance (ANOVA) followed by Tukey comparison test using GraphPad Prism (version 8.00) as the software program. Significant differences were considered when *p* < 0.05.

## 3. Results

### 3.1. Effects on Liver Enzymes

Acute i.p. injection of CCl_4_ significantly (*p* < 0.05) induced ca. 2.5- and 3-fold increases in serum AST and ALT levels, respectively, compared to the negative control group. However, treatment of animals with naringenin at both doses (20 and 40 mg/kg) significantly (*p* < 0.05) decreased liver enzymes as compared to the CCl_4_-treated group. The minimizing effect of naringenin at both doses on both liver enzymes was comparable to the standard drug, silymarin (Table 1). 

### 3.2. Effects on Kidney-Related Parameters

As shown in Table 1, CCl_4_ i.p. injection showed a significant (*p* < 0.05) and marked elevation in serum levels of creatinine, urea and uric acid as compared to the negative control group. Treatment with naringenin at both doses (20 and 40 mg/kg) significantly (*p* < 0.05) decreased these renal parameters as compared to the CCl_4_-treated group, and the effect was comparable to that of silymarin. It is worth noting that naringenin at both doses (20 and 40 mg/kg) decreased serum levels of creatinine and uric acid at levels that were not significantly different (*p* < 0.05) from the normal values.

### 3.3. Histopathological Examination

**Figure 1:**
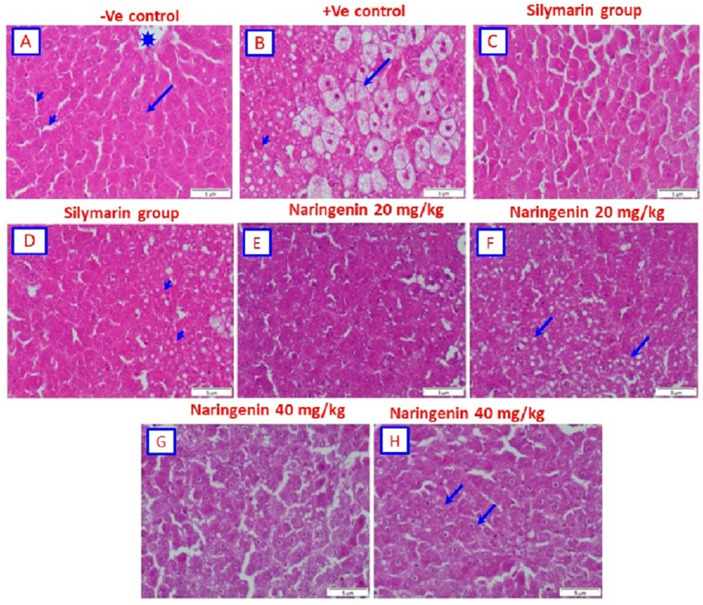
Photomicrograph of the liver tissues using hematoxylin and eosin (H&E) staining: (**A**) Negative control rats showing the normal structure of the hepatic lobule. The central vein (asterisk) is at the center of the lobule, which is surrounded by cords of hepatocytes (arrow). The hepatic sinusoids are shown between the strands of hepatocytes (arrowheads). (**B**) Rat given CCl_4_ (positive control) showing large vacuoles in the cytoplasm with displacement of nuclei. The cytoplasm of the hepatocytes (arrowheads) has an even pale appearance, similar to ground glass. The hepatocytes undergo hydropic degeneration and become swollen and vacuolated (arrow), while some hepatocytes show an accumulation of eosinophilic material known as Mallory’s hyaline. (**C**) Rat given CCl_4_ and treated with silymarin showing hepatocytes that appear as more or less normal. (**D**) Rat given CCl_4_ and treated with silymarin showing hepatocytes associated with small vacuoles. (**E**) Rat given CCl_4_ and treated with (20 mg/kg) naringenin showing hepatocytes that appear as more or less normal. (**F**) Rat given CCl_4_ and treated with (20 mg/kg) naringenin showing hepatocytes associated with small vacuoles. (**G**) Rat given CCl_4_ and treated with (40 mg/kg) naringenin showing hepatocytes that appear as more or less normal. (**H**) Eat given CCl_4_ and treated with (40 mg/kg) naringenin showing several hepatocytes associated with small vacuoles (H&E; scale bar: 5 µm).

Histological study of sections from the control rat liver revealed a normal architecture of hepatic lobules. The central vein was at the center of each lobule and is surrounded by cords of hepatocytes. The hepatic sinusoids were observed between the strands of hepatocytes (Figure 1A). Microscopic examination of livers from rats administered CCl_4_ alone showed large vacuoles in the cytoplasm with displacement of nuclei. The cytoplasm of the hepatocytes had an even pale appearance, similar to ground glass. The hepatocytes underwent hydropic degeneration and became swollen and vacuolated, with some hepatocytes exhibiting an accumulation of eosinophilic materials known as Mallory’s hyaline (Figure 1B). For rats that were administered CCl_4_ and treated with silymarin as the positive drug control, the histopathological investigation showed that the hepatocytes appeared more or less close to normal (Figure 1C). In addition, sections showed hepatocytes associated with small vacuoles (Figure 1D). Micrographs from sections of the livers of rats given CCl_4_ and treated with naringenin (20 mg/kg) showed that hepatocytes appeared more or less normal (Figure 1E). On the other hand, hepatocytes were found to be associated with small vacuoles (Figure 1F). Rats given CCl_4_ and treated with 40 mg/kg of naringenin showed that hepatocytes appeared more or less normal (Figure 1G). However, in some sections, several hepatocytes associated with small vacuoles were found (Figure 1H).

**Figure 2:**
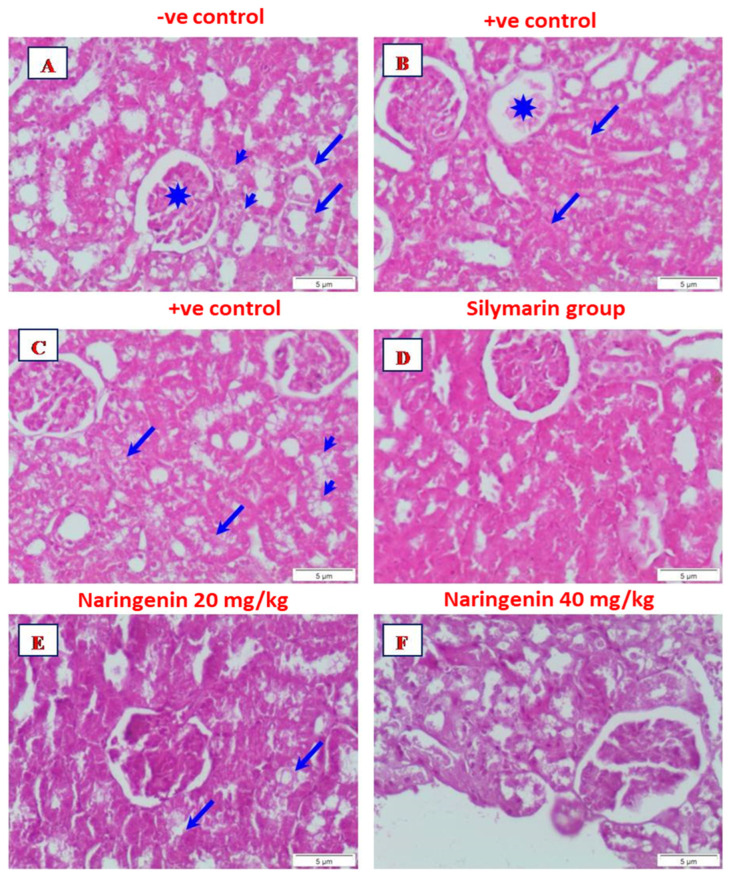
Micrograph from a section of the kidney of (**A**) control rat showing normal structure of renal corpuscle (asterisk) and renal tubules, distal (arrows), and proximal (arrowheads) convoluted tubules; (**B**) rat given CCl_4_ showing partially degenerated glomerulus (asterisk). Focal of interstitial hemorrhage is seen (arrows): (**C**) rat given CCl_4_ showing necrosis of the renal tubules (arrows). Some renal tubules showed complete degeneration (arrowheads): (**D**) rat given CCl_4_ and treated with silymarin showing the renal corpuscle and renal tubules appeared nearly to that of normal control; (**E**) rat given CCl_4_ and treated with naringenin (20 mg/kg) showed the renal corpuscle and renal tubules appeared nearly to normal control. Noticed degenerative renal tubules (arrows): (**F**) rat given CCl_4_ and treated with naringenin (40 mg/kg) showed the renal corpuscle and renal tubules appeared more or less like normal (H&E, Scale bar: 5 µm).

Microscopic analysis of the kidney sections of rats in the control group exhibited normal structures of the renal corpuscle and the renal, distal and proximal convoluted tubules (Figure 2A). Histopathological investigation of the kidney sections from rats given CCl_4_ alone showed partial degeneration of the glomeruli. In addition, areas of interstitial hemorrhage were observed (Figure 2B). On the other hand, necrosis of the renal tubules was observed, while other renal tubules showed complete degeneration (Figure 2C). FOR rats given CCl_4_ and treated with silymarin, the renal corpuscle and renal tubules appeared similar to the normal control (Figure 2D). Examination of kidney sections of rats given CCl_4_ and treated with 20 mg/kg of naringenin indicated that renal corpuscles and renal tubules appeared comparable to the normal control, although with some degenerative renal tubules (Figure 2E). For rats given CCl_4_ and treated with 40 mg/kg of naringenin, the renal corpuscles and renal tubules appeared more or less normal (Figure 2F).

### 3.4. Bcl-2 Immuno-Histochemical Expression

**Figure 3:**
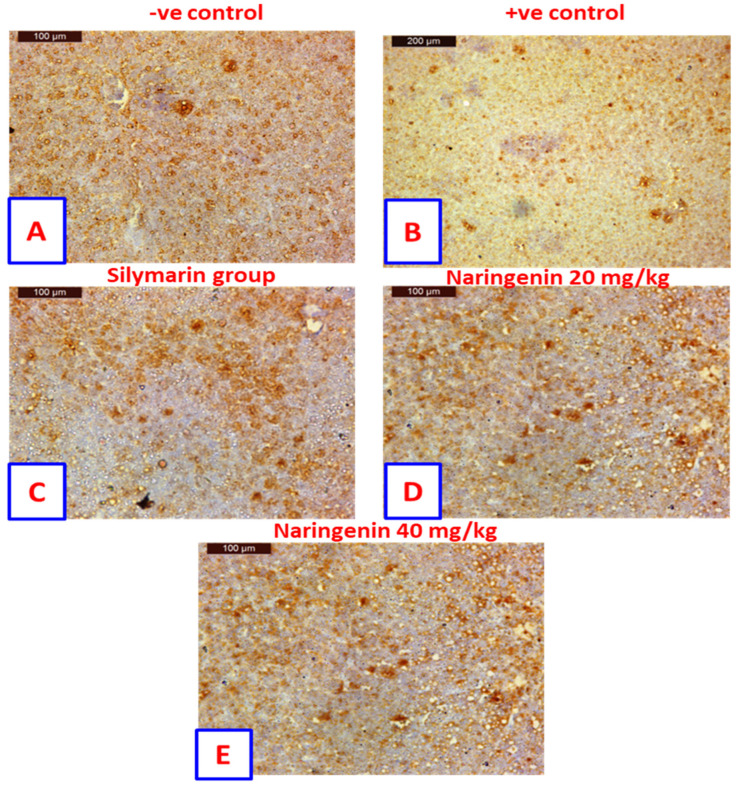
Sections from liver showing immunohistochemical expression of Bcl−2 in the hepatocytes of (**A**) control rats. Positively stained hepatocytes are contrasted with nonstaining nuclei; (**B**) positive control rat exhibit negatively stained hepatocytes are contrasted with nonstaining nuclei; (**C**) rat treated with silymarin drug indicated more positively stained hepatocytes are contrasted with nonstaining nuclei; (**D**) rat treated with 20 mg/kg of naringenin. Positively stained hepatocytes are contrasted with nonstaining nuclei: (**E**) rat treated with 40 mg/kg of naringenin, positively stained hepatocytes appearing contrasted with nonstaining nuclei (Immunohistochemical expression of Bcl−2, Scale bar: µm).

Liver sections showed Bcl-2 immuno-histochemical expression in the hepatocytes of negative control rats, showing further positively stained hepatocytes that contrasted with non-staining nuclei (Figure 3A). The positive control rats showed negatively stained hepatocytes contrasted with non-staining nuclei (Figure 3B). In rats treated with silymarin, drug staining indicated more positivity in hepatocytes that were associated with non-staining nuclei (Figure 3C). In contrast, rats treated with 20 mg/kg of naringenin showed positively stained hepatocytes contrasted with non-staining nuclei (Figure 3D). Likewise, rats treated with 40 mg/kg of naringenin also showed positively stained hepatocytes contrasted with non-staining nuclei (Figure 3E).

**Figure 4:**
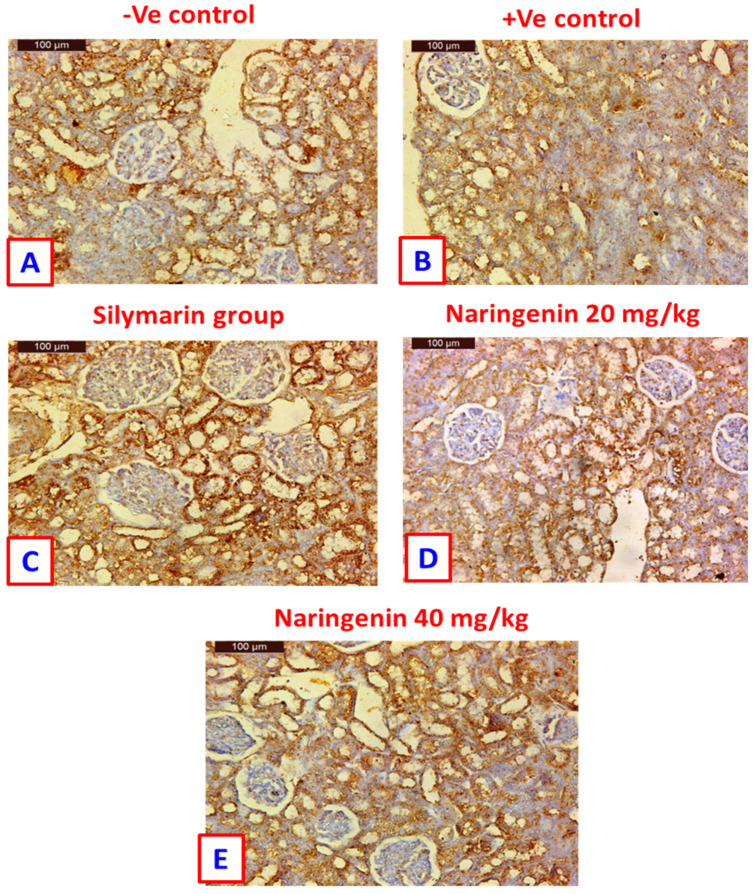
Immuno-histochemical detection of Bcl-2 in kidney tissues: (**A**) control rat showing positive staining in the renal tubules, contrasted with non-staining glomeruli; (**B**) positive control rat showing negative staining in the damaged tubules, while the healthy one shows positive staining; (**C**) rat treated with silymarin drug showing high immuno-histochemical expression in the renal tubules as compared to the positive control; (**D**) kidney sample of rat treated with 20 mg/kg of naringenin showing positive immuno-histochemical expression in the renal tubules and negative expression in the glomeruli; (**E**) rat treated with naringenin (40 mg/kg) showing greater immuno-histochemical expression of Bcl-2 than in the rat treated with naringenin (20 mg/kg) (immuno-histochemical expression of Bcl-2; scale bar: µm).

Sections from kidneys of control rats showed Bcl-2 immuno-histochemical expression in the renal tubules and non-staining glomeruli (Figure 4A). For rats from the positive control group, negative staining was observed in the damaged tubules, while the healthy rats showed positive staining (Figure 4B). Rats treated with silymarin showed highly immuno-histochemical expression in the renal tubules as compared with positive controls (Figure 4C). Kidney samples of rats treated with 20 mg/kg of naringenin showed positive immuno-histochemical expression in the renal tubules and negative expression in the glomeruli (Figure 4D). On the other hand, rats treated with the 40 mg/kg dose of naringenin showed greater immune-histochemical expression of Bcl-2 than rats treated with 20 mg/kg (Figure 4E).

In parallel, quantitative estimation of BCl-2 immuno-expression in hepatic tissue revealed that CCl_4_ in positive control rats significantly reduced (*p* < 0.05) the area of BCl-2 expression (by about 5-fold) as compared to the negative control group (Supplementary Figure S1). However, treatment of rats with silymarin and naringenin at 20 and 40 mg/kg showed increased BCl-2 expression to 18.2%, 22.4% and 23.2%, respectively; as compared to the CCl_4_-positive control value (8.7%). It is worth noting that naringenin at both doses increased the expression of BCl-2 by more than that of the standard hepatoprotectant, silymarin. Similarly, the area of BCl-2 immune-expression in the renal tissue was decreased in the CCl_4_-treated rats and again enhanced in the treatment groups with silymarin and naringenin at 20 and 40 mg/kg to 33.3%, 24.7% and 28.4%, respectively (Supplementary Figure S2). 

### 3.5. Serum Metabolite Profiling in CCl_4_-Induced Rat Livers and with Treatments as Analyzed Using GC–MS

Metabolite profiling by GC–MS was coupled with multivariate data analysis to investigate the therapeutic systemic effects of naringenin in rats, as well as for the discovery of biomarkers for illness progression. Metabolite profiling led the identification of a total of 82 low molecular weight metabolites. Individual component identities, retention indices (RI) and retention times (RT) are included in Supplementary Table S1. Furthermore, Supplementary Figure S3 shows typical chromatograms of all analyzed serum specimens from each animal group.

### 3.6. Multivariate Analysis of Collected Datasets from All Treatment Groups

**Figure 5:**
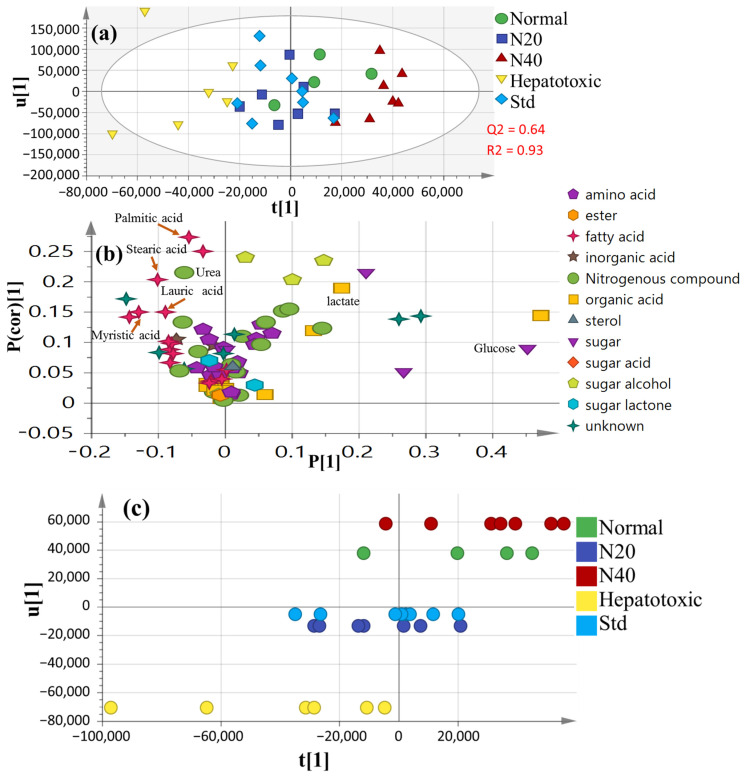
Supervised OPLS dataset for all groups (*n* = 6): (**a**) OPLS-DA score plot; (**b**) The corresponding loading plot demonstrating the covariance P(1) versus the correlation P(cor)(1); (**c**) OPLS class inner relation with 40 mg naringenin (colored in red), showing a close relation to the normal untreated group (colored in green). N20: naringenin at 20 mg/kg; N40: naringenin at 40 mg/kg; Std: standard drug (silymarin).

Unsupervised PCA (principal component analysis) was unable to distinguish between the metabolite profiles of these various groups. Thus, a supervised OPLS-DA design was also employed for greater distinction, while the other sources of variance were down-weighted [30], demonstrating model validity through the covered variance R2 value (93%) and prediction power Q2 value (64%) (Figure 5a). Figure 5b shows the loading plot generated from the OPLS-DA score plot. The metabolites leading to the group’s distinction encompassed glucose, urea and some fatty acids (FAs), i.e., lauric, myristic, palmitic and stearic acids, as depicted in the corresponding loading plot. 

Serum metabolite profiles in CCl_4_-intoxicated rats differed the most from the normal group (Figure 5a), suggesting that CCl_4_ -induced liver injury disrupted endogenous serum metabolism relative to the normal condition. The disrupted metabolite profiling reported in the CCl_4_ model group was recovered after treatment with naringenin at a high dosage (40 mg/kg) to a range near that of the normal healthy group. The OPLS-DA class inner relationship dataset (Figure 5c) demonstrated that naringenin at the higher dose of 40 mg was the furthest group from the CCl_4_-intoxicated group, with considerable discrepancies in their metabolite profiles. 

### 3.7. OPLS-DA Analysis of Collected Dataset from CCl4-Induced Liver Injury versus Normal Control

**Figure 6:**
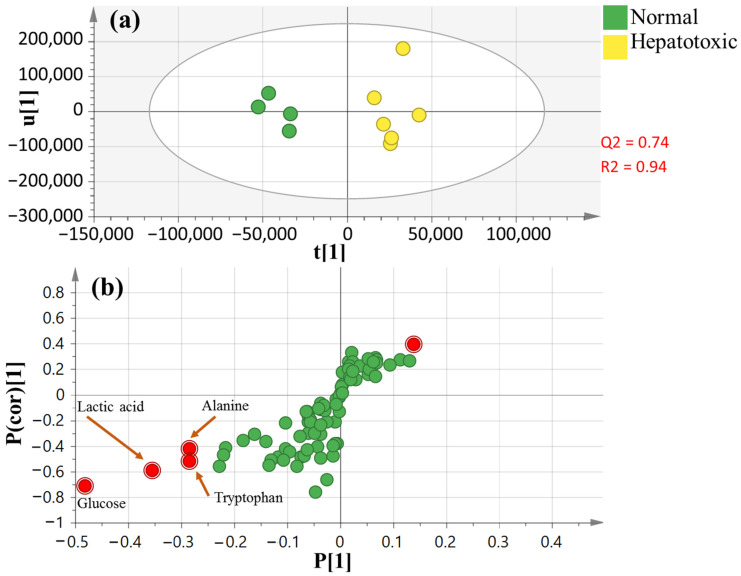
OPLS dataset of healthy normal and CCl_4_-intoxicated groups (*n* = 6): (**a**) OPLS-DA score plot; (**b**) loading S-plot.

By comparing normal control vs. CCl_4_-treated rats, the OPLS-DA model and its associated loading S-plot (Figure 6a,b) were exploited to discover metabolite indicators of the CCL_4_-intoxicated group. Higher R^2^ (0.94 for the total variance) and Q^2^ (0.74 with prediction goodness) values were observed in all groups (Figure 6a). Lactic acid, glucose, alanine and tryptophan were observed at lower levels in the CCl_4_-intoxicated group relative to the normal healthy untreated control group.

### 3.8. OPLS-DA Analysis of Collected Dataset from Normal Control, CCl_4_-Intoxicated and Naringenin (40 mg/kg) Groups

**Figure 7:**
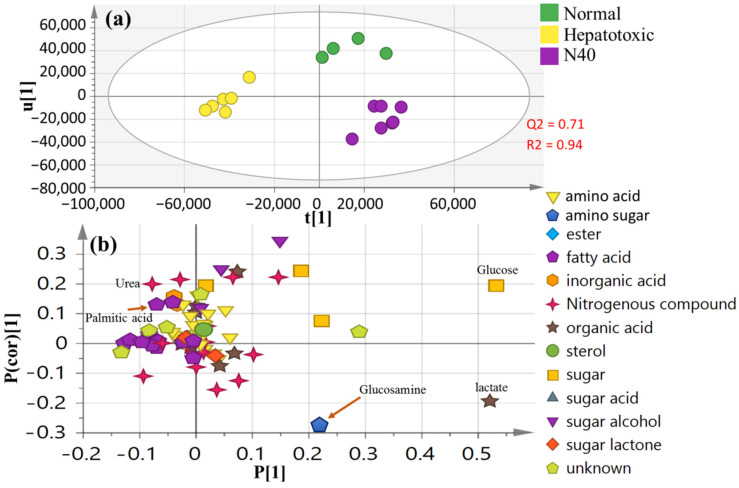
Supervised OPLS dataset of healthy normal, intoxicated and naringenin at 40 mg/kg groups (*n* = 6): (**a**) OPLS-DA score plot for N40 (naringenin at 40 mg/kg); (**b**) the corresponding loading plot.

To further investigate whether naringenin can return CCl_4_-induced toxicity to normal again, an OPLS-DA model including CCl_4_-intoxicated, healthy control and naringenin (at higher dose 40 mg/kg) groups was employed (Figure 7a). The score plot revealed segregation between the CCl_4_-intoxicated group and the normal healthy group, while naringenin at 40 mg/kg was clustered closer to the normal group. The related loading plot as indicated in Figure 7b revealed elevated glucosamine levels in naringenin in the 40-mg/kg-treated rat group.

## 4. Discussion

CCl_4_-induced acute hepatic injury is a well-established model for screening the hepatoprotective effects of new drugs [31,32]. Experimental liver damage induced by CCl_4_ and pathological lesions resembles the clinical case of viral hepatitis. Hepatotoxicity due to CCl_4_ results in leakage of the intracellular metabolic enzymes, causing tissue malfunction. CCl_4_ is also the best considered chemical to investigate chemical-induced acute liver injury in rats. This hepatotoxin is an extensively used industrial solvent that is highly lipid-soluble and stimulates the evolution of free radicals, resulting in damage to different tissues, including the liver. Typical markers used for evaluating the extent of hepatic damage induced by CCl_4_ are obtained through measurements of the serum levels of the cytoplasmic enzymes (ALT and AST) that leak from the damaged hepatocytes into the blood, indicating the presence of cellular infiltration, centrilobular necrosis and ballooning degeneration [33]. In the present study, a single i.p. injection of CCl_4_ induced marked elevation of liver enzymes, indicating the establishment of hepatotoxicity, as previously reported [34]. In parallel, histopathological examination confirmed the biochemical analysis and showed an abnormal liver architecture with the presence of large vacuoles in the cytoplasm of hepatocytes. The cytoplasm appeared pale with displacement of nuclei. The hepatocytes underwent hydropic degeneration and were swollen, with some hepatocytes exhibiting an accumulation of eosinophilic materials known as Mallory’s hyaline, as in previous studies [32,35].

Likewise, CCl_4_ induces major injuries in the kidneys due to the formation of free radicals, as well as alterations in the antioxidant system leading to increased lipid peroxidation [36]. It was shown that CCl_4_ affects the renal cortex, which contains the key enzyme implicated in CCl_4_-induced nephrotoxicity, the cytochrome P-450 [37]. In the current study, CCl_4_ injection induced disruption in renal function, as mirrored by increased levels of serum creatinine, urea and uric acid above the normal values, and in accordance with previous studies [38]. Moreover, CCl_4_ markedly induced histopathological alterations and interfered with the normal renal tissue architecture in the form of completed degeneration, necrosis of renal tubules and partial degeneration of glomeruli, as well as the appearance of areas of interstitial hemorrhage, as reported previously [39,40]. 

Apoptosis involves a programmed cascade of enzymatic events that achieve cell death for the clearance of infiltrating inflammatory cytokines in an attempt to overcome tissue toxicity. However, inappropriate stimulation of apoptosis, including activation of caspases, may further potentiate renal toxicity by exerting damage to tubular cells [41]. Caspases are involved in apoptosis, whereas initiator caspase (caspase-3) activates the killer caspase (caspase-9) downstream of the key structural protein B-cell lymphoma 2 (Bcl-2) and plays a key role in the accomplishment of apoptosis [42]. Similarly in hepatic tissue, CCl_4_ triggers apoptosis by releasing large amounts of reactive oxygen species. The mitochondrial pathway that involves a balancing act between the pro- and antiapoptotic Bax/Bcl-2 family proteins plays an important role in CCl_4_-induced apoptosis [43]. In parallel with previous studies, acute injection of CCl_4_ in the present study inhibited Bcl-2 expression in renal and hepatic tissues [44,45].

Naringenin is a well-known anti-inflammation compound due to its modulatory effect on pro- and anti-inflammatory cytokines [46,47,48]. Additionally, several documented studies showed the significant potential of naringenin to induce apoptosis through the downregulation of Akt and caspase-3 activation in in vivo and in vitro studies [49,50,51].

Treatment with naringenin in the present study efficiently improved the hepatic and renal injuries caused by acute injection of CCl_4_. It decreased the elevated liver enzymes (AST and ALT) to levels comparable to the standard hepatoprotective drug, silymarin, indicating the ability of naringenin to conserve the membrane integrity of hepatocytes of CCl_4_-intoxicated rats, probably by decreasing the production of CCl_4_, a highly reactive metabolite. Naringenin at both doses normalized the increased serum creatinine and uric acid levels and decreased urea levels as compared to the CCl_4_-intoxicated group. The hepato- and renoprotective effects of naringenin were further confirmed by histopathological examination of the liver and kidney tissues. Pretreatment of rats with naringenin at both doses minimized the severity of CCl_4_-induced liver damage and improved the architecture of hepatic and renal tissues in a dose-dependent manner. Additionally, the immuno-histochemical investigation showed that naringenin successfully enhanced Bcl-2 expression in liver and kidney tissues at the high dose (40 mg/kg), showing the highest level of immune staining of hepatocytes and renal tubules. Additionally, naringenin at the high dose (40 mg/kg) showed a higher percentage of BCl-2 expression areas in the renal tissue as compared to the lower dose treatment group. Naringenin at both doses (20 and 40 mg/kg) presented higher levels of BCl-2 expression areas in hepatic tissue as compared to the standard drug, silymarin.

In parallel, the hepatoprotective effect was previously described for naringenin in different animal models. Naringenin could attenuate experimentally induced non-alcoholic fatty liver disease in mice via its anti-inflammatory activity [8] and protect against doxorubicin-induced liver dysfunction in rats probably via its antioxidant properties [52]. *Carissa carandas* extract, which contains naringen, a 7-*O*-glycoside of naringenin, as one of its major constituents showed a hepatoprotective effect against CCl_4_-induced hepatotoxicity by adjusting liver enzymes (ALT, AST, ALP and GGT) and bilirubin, as well as amelioration of histopathological features of liver damage induced by CCl_4_, such as neutrophil infiltration, severe necrosis and hydropic degeneration [53].

However, research is still needed to validate the use of naringenin as a potential candidate for renal and liver function improvements based on mechanistic studies [4]. Hence, a GC–MS-based metabolomic analysis was performed to investigate the metabolic changes in the CCl_4_-induced liver injury model and to demonstrate the effectiveness of naringenin as a hepatoprotective agent, as shown by restoring metabolites that were perturbed by CCl_4_ close to normal levels (Figure 7). Metabolomics data reflecting the most downstream metabolite information in cellular processes were monitored as a direct response to pathophysiological changes. In the present study, levels of urea, palmitic acid, stearic acid, lauric acid and myristic acid were significantly increased after CCl_4_ exposure, whereas decreased glucose, lactate tryptophan and alanine was observed. Naringenin appeared to ameliorate the liver injuries in the CCl_4_-induced rats by restoring the complex responses from multiple interconnected metabolic pathways (Figure 7).

Regarding lipids, there were significantly elevated levels of palmitic acid, lauric acid, stearic acid and myristic acid-related metabolites. In vertebrates, the liver is the major organ for FA production and metabolism. It is predictable that any damage to the liver will cause a change in the balance of FA levels, including free and esterified FAs [54]. A high quantity of free fatty acids in the body can cause a considerable amount of lipids to accumulate in hepatic cells, causing cell membrane, mitochondria and lysosome damage [55]. 

Palmitic acid and lauric acid are saturated FAs that shown at obvious levels in human serum, urine and cerebrospinal fluid. Our analysis indicated significant increases in levels of these FAs in serum samples from rats in the CCl_4_ group as a consequence of upregulated de novo lipid synthesis. Additionally, increased oxidative stress is liable to cause an extra increase in lauric acid [56].

Interestingly, palmitic acid is a significant compound in the fatty acid biosynthesis pathway, and it was found to be a biomarker of liver injury in this study. Palmitic acid expression was aberrant in our study, indicating that fatty acid biosynthesis was significantly disrupted. Furthermore, the liver plays a significant role in fatty acid metabolism because it absorbs a high amount of free FAs (FFAs), accessing the splanchnic bed through the portal vein [57], whereas the non-hepatic splanchnic bed absorbs only a tiny fraction of FFAs. When the liver’s FA metabolism is insufficient, FFA levels in the blood and uptake by the liver increase, resulting in lipid deposition in liver cells and cytotoxicity [58]. Under normal conditions, mitochondria may deconstruct large amounts of FAs in cells and make ATP via FA beta-oxidation production, despite the electron respiratory chain leading to extreme production of reactive oxygen species (ROS) [44]. Oversupply of ROS damages the mitochondrial structure and function, resulting in aggregate cellular oxidative stress and initiating FA catabolism impairment [59,60]. In addition, palmitic acid in CCl_4_ rats is a significant element of FFAs in the blood. However, FA accumulation induces cellular damage linked to the generation of ROS, as well as increases in apoptosis and necrosis indicators and a decrease in albumin production [61]. In accordance with our results, previous studies reported that NAFLD patients have high levels of saturated FAs (SFAs) and low levels of polyunsaturated FAs (PUFA) [62]. 

Concerning the increase in myristic acid, this long-chain FA has a strong effect on hepatic cells, causing cellular stress and steatosis [63]. According to other research, it also raises the risk of cardiovascular disease [64]. It can be argued that naringenin mainly improves liver damage by managing FA metabolism abnormalities, as evidenced by decrease in FAs level close to normal groups.

With regards to liver intoxication effects on polar metabolites detected in serum samples, GC–MS showed that the serum levels of alanine and tryptophan were disturbed in the CCl_4_ group, suggestive for alterations in amino acid metabolism (Figure 6). The liver is an essential organ for modulating amino acid metabolism, and any disorders in the liver could result in amino acid disruption [65]. The decreased alanine and tryptophan levels after CCl_4_ administration were likely related to the downregulation of alanine and tryptophan biosynthesis. Essential amino acids serve an important role in regulating the body’s energy metabolism and protein synthesis [66], while alterations in their levels might affect the metabolic and functional status of the body, leading to energy metabolism disorders in rats [67]. Moreover, this finding could be a result of satisfying the rising demand for proteins and also as a result of increasing gluconeogenesis in CCl_4_-treated rats to fulfill the energy deficit [68]. 

Additionally, in parallel with the current findings, only a few reports have revealed the renoprotective effects of naringenin. It protected against sepsis-induced acute kidney injury in a previous study [69]. When combined with quercetin, naringenin ameliorated reno- and hepatotoxicity in rats [70]. Naringenin also attenuated the nephrotoxicity triggered by oxytetracycline via it antioxidant activities [71]. The decreased levels of serum lactate and alanine in the CCl_4_ group in our study might demonstrate both nephrotoxicity and renal reabsorption impairment [72].

Parallel with those amino acid metabolism disorders, urea levels increased in the CCl_4_ group, further suggesting abnormal liver and kidney functions. Urea is the main final catabolic product of amino acids; an elevation in its blood levels could indicate impaired kidney function [73].

Glucose was identified as one of the key metabolites affected in acute liver injury models, which was lower in the CCl4 group compared to the control normal group. The decrease was in line with the previous report [74,75] and attributed to enhanced energy demands and glycolysis. In contrast, naringenin treatment elevated serum glucose levels back to normal, inferring downregulated glycolysis or enhanced gluconeogenesis.

Moreover, the decrease in glucose levels suggests that liver disorders, such as fibrosis, are linked to a disruption in carbohydrate metabolism. The ability for insulin inactivation in the liver is impeded in the process of liver disease, and as a result insulin levels in the blood are dramatically elevated [76], ultimately leading to lower glucose levels [77]. The major raw materials available for energy use in the liver are saccharides such as glucose [78]. The glucose content in the serum samples of the model group was downregulated in this study, which was most likely due to changes in glycometabolism. The naringenin intervention significantly increased glucose levels, indicating that it can regulate energy metabolism.

Glucosamine is an amino sugar and a prominent precursor in the biochemical synthesis of glycosylated proteins and lipids. In this study, a relative increase in glucosamine was observed in the naringenin group as compared to the model control group. The previous studies [79] reported that glucosamine had a protective effect against carbon-tetrachloride-induced liver damage in mice by decreasing serum AST and ALT activities and MDA formation.

## 5. Conclusions

The present results showed that *Citrus* naringenin effectively protects against CCl_4_-induced hepatic and renal injuries in rats by reducing serum ALT and AST levels and decreasing urea, creatine and uric acid levels. It also improved histopathological changes induced in liver and kidney tissues in a dose-dependent manner. To shed light on the mechanisms of the hepato- and renoprotective effects of naringenin, a GC–MS-based metabolomics method was successfully applied. The metabolomics analysis revealed that naringenin may be able to help fix the impaired metabolic pathways, such as FA metabolism, amino acid metabolism, energy metabolism and kidney metabolism (i.e., alanine and lactic acids). Therefore, naringenin may be a potential treatment for CCL4-induced hepatic and renal damage. However, further studies are needed to corroborate our findings in other liver intoxication models or ideally in clinical studies.

## Figures and Tables

**Table 1:** Effects of naringenin on the liver and kidney markers in CCl_4_-administered rats.

Treatment	Liver Enzymes		Kidney Markers		
	AST (U/L)	ALT (U/L)	Serum Creatinine (mg/dL)	Urea (mg/dL)	Uric Acid (mg/dL)
Negative control	95.37 ± 7.13 ^b^	46.51 ± 1.68 ^b^	0.89 ± 0.037 ^b^	10.22 ± 0.38 ^b^	7.49 ± 0.25 ^b^
Positive control	250.30 ± 12.25 ^a^	137.50 ± 5.82 ^a^	1.71 ± 0.11 ^a^	24.56 ± 0.79 ^a^	12.43 ± 0.22 ^a^
Silymarin (50 mg/kg)	157.10 ± 6.58 ^a,b^	100.41 ± 7.76 ^a,b^	0.85 ± 0.01 ^b^	11.07 ± 0.47 ^b^	6.97 ± 0.24 ^b^
Naringenin (20 mg/kg)	162.90 ± 5.35 ^a,b^	115.50 ± 1.43 ^a,b^	0.77 ± 0.02 ^b^	15.8 ± 0.73 ^a,b^	7.89 ± 0.22 ^b^
Naringenin (40 mg/kg)	152.60 ± 12.82 ^a,b^	108.10 ± 4.64 ^a,b^	0.73 ± 0.02 ^b^	14.63 ± 0.32 ^a,b^	7.46 ± 0.14 ^b^

Each value represents the mean ± SEM (*n* = 6). Statistical analysis was achieved via one-way ANOVA followed by Tukey’s post hoc test. ^a^ Significantly different form negative control. ^b^ Significantly different from positive control (CCl_4_) at *p* < 0.05.

## Data Availability

Data are contained within the article.

## Supplementary Materials

The following supporting information can be downloaded at: https://www.mdpi.com/2072-6643/14/4/841/s1, Figure S1: Area of BCl2 immnuohistochemical expression in hepatic tissue measured by the image analysis system as area percent. Data are expressed as Mean ± SE (*n* = 6). Statistical analysis was carried out by one-way ANOVA using Tukey as post-hoc test.; Figure S2: Area of BCl2 immnuohistochemical expression in renal tissue measured by the image analysis system as area percent. Data are expressed as Mean ± SE (*n* = 6). Statistical analysis was carried out by one-way ANOVA using Tukey as post-hoc test.; Figure S3: The representative chromatograms of serum samples from different animal groups; Table S1: Relative percentile of silylated primary metabolites in sera from all groups using SPME-GC–MS (*n* = 6), results are presented as average ± (std. deviation).
