# Correction: Ammar et al. Protective Effects of Naringenin from *Citrus sinensis* (var. Valencia) Peels against CCl_4_-Induced Hepatic and Renal Injuries in Rats Assessed by Metabolomics, Histological and Biochemical Analyses. *Nutrients* 2022, *14*, 841

**DOI:** 10.3390/nu16030394

**Published:** 2024-01-30

**Authors:** Naglaa M. Ammar, Heba A. Hassan, Heba M. I. Abdallah, Sherif M. Afifi, Abdelbaset M. Elgamal, Abdel Razik H. Farrag, Abd El-Nasser G. El-Gendy, Mohamed A. Farag, Abdelsamed I. Elshamy

**Affiliations:** 1 Therapeutic Chemistry Department, Pharmaceutical and Drugs Research Institute, National Research Centre, Giza 12622, Egypt; nm.ammar@nrc.sci.eg (N.M.A.); ha.el-saud@nrc.sci.eg (H.A.H.); 2 Pharmacology Department, Medical Research and Clinical Studies Institute, National Research Centre, Giza 12622, Egypt; heba21_5@yahoo.com; 3 Pharmacognosy Department, Faculty of Pharmacy, University of Sadat City, Sadat City 32897, Egypt; 4 Chemistry of Microbial and Natural Products Department, Pharmaceutical and Drugs Research Institute, National Research Centre, Giza 12622, Egypt; algamalgene@yahoo.com; 5 Department of Pathology, Medical Research and Clinical Studies Institute, National Research Centre, Giza 12622, Egypt; ar.hussein@nrc.sci.eg; 6 Medicinal and Aromatic Plants Research Department, Pharmaceutical and Drugs Research Institute, National Research Centre, Cairo 12622, Egypt; aggundy_5@yahoo.com; 7 Pharmacognosy Department, College of Pharmacy, Cairo University, Cairo 11562, Egypt; mfarag73@yahoo.com; 8 School of Forestry and Biotechnology, Zhejiang A&F University, Hangzhou 311300, China; 9 Chemistry of Natural Compounds Department, Pharmaceutical and Drugs Research Institute, National Research Centre, Giza 12622, Egypt

## Error in Figure

In the original publication [1], there was a mistake in Figures 2 and 3. The authors uploaded the wrong image during the final proofreading. In the original version, Figure 2 displays the micrograph from a section of the kidney. However, Figure 3 displays sections from the liver showing the immunohistochemical expression of Bcl-2 in the hepatocytes. There are duplications in the published Figure 2: Figure 2C,E as well as 2D and 2F are same. Also, there are duplications in the published Figure 3: Figure 3D,E are same. The corrected Figure 2 and Figure 3 appear below. The authors apologize for any inconvenience caused and state that the scientific conclusions are unaffected. This correction was approved by the Academic Editor. The original publication has also been updated.

## Figures and Tables

**Figure 2:**
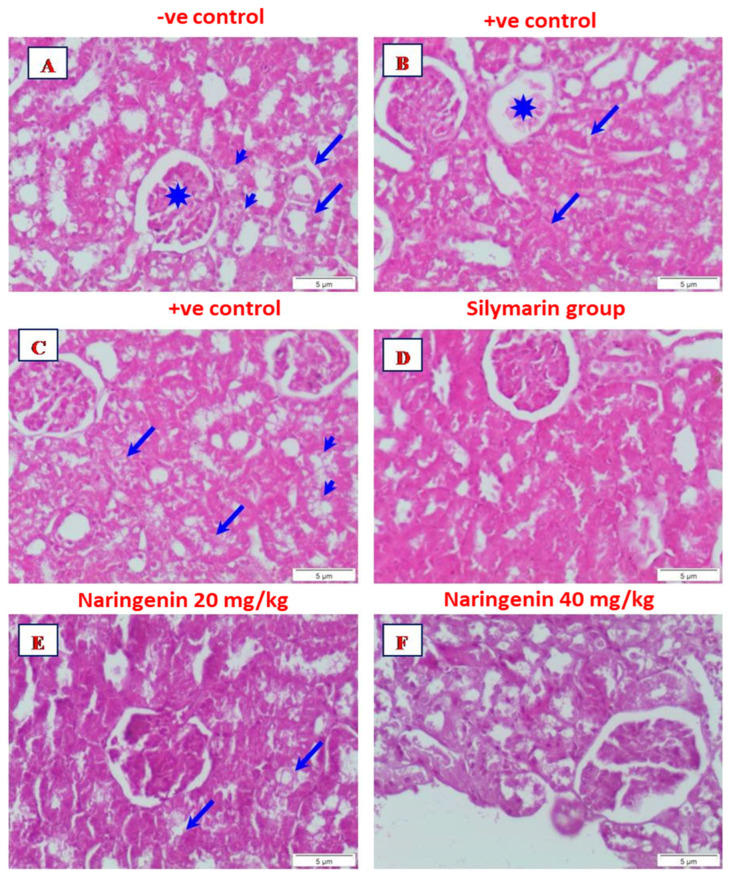
Micrograph from a section of the kidney of (**A**) control rat showing normal structure of renal corpuscle (asterisk) and renal tubules, distal (arrows), and proximal (arrowheads) convoluted tubules; (**B**) rat given CCl_4_ showing partially degenerated glomerulus (asterisk). Focal of interstitial hemorrhage is seen (arrows): (**C**) rat given CCl_4_ showing necrosis of the renal tubules (arrows). Some renal tubules showed complete degeneration (arrowheads): (**D**) rat given CCl_4_ and treated with silymarin showing the renal corpuscle and renal tubules appeared nearly to that of normal control; (**E**) rat given CCl_4_ and treated with naringenin (20 mg/kg) showed the renal corpuscle and renal tubules appeared nearly to normal control. Noticed degenerative renal tubules (arrows): (**F**) rat given CCl_4_ and treated with naringenin (40 mg/kg) showed the renal corpuscle and renal tubules appeared more or less like normal (H&E, Scale bar: 5 µm).

**Figure 3:**
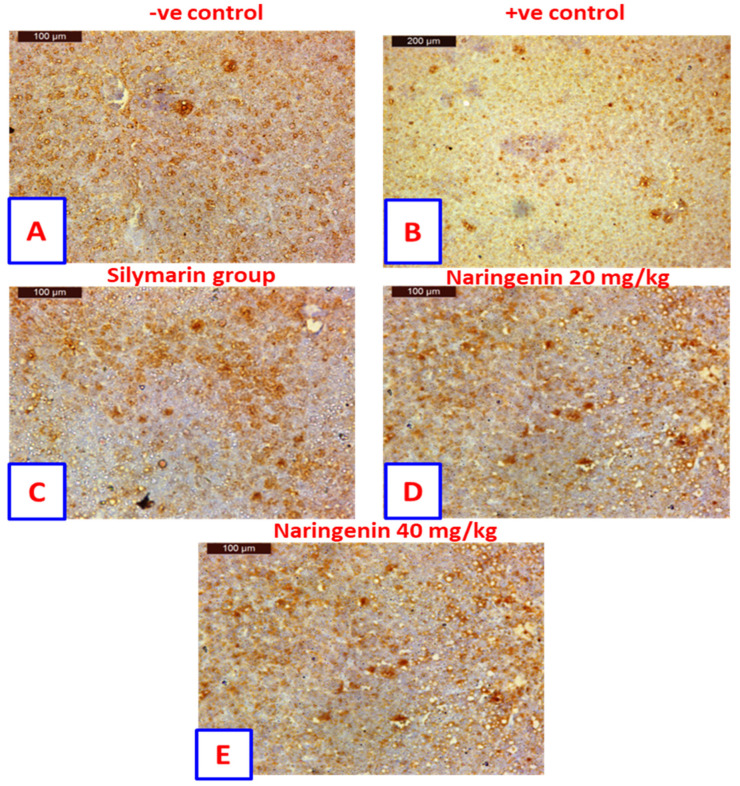
Sections from liver showing immunohistochemical expression of Bcl−2 in the hepatocytes of (**A**) control rats. Positively stained hepatocytes are contrasted with nonstaining nuclei; (**B**) positive control rat exhibit negatively stained hepatocytes are contrasted with nonstaining nuclei; (**C**) rat treated with silymarin drug indicated more positively stained hepatocytes are contrasted with nonstaining nuclei; (**D**) rat treated with 20 mg/kg of naringenin. Positively stained hepatocytes are contrasted with nonstaining nuclei: (**E**) rat treated with 40 mg/kg of naringenin, positively stained hepatocytes appearing contrasted with nonstaining nuclei (Immunohistochemical expression of Bcl−2, Scale bar: µm).
