# Supplementary material for: Protective Effects of Naringenin from Citrus sinensis (var. Valencia) Peels against CCl4-Induced Hepatic and Renal Injuries in Rats Assessed by Metabolomics, Histological and Biochemical Analyses
Source: Nutrients. 2022 Feb 17;14(4):841. doi: 10.3390/nu14040841 (PMC8924893; doi:10.3390/nu14040841)
Supplement: Supplementary file 1 [file nutrients-14-00841-s001.zip › nutrients-1542567-supplementary.pdf]

Supplementary Materials

Protective Effects of Naringenin from *Citrus sinensis* (var. Valencia) Peels against CCl4-Induced Hepatic and Renal Injuries in Rats Assessed by Metabolomics, Histological and Biochemical Analyses

Naglaa M. Ammar [1], Heba A. Hassan [1], Heba M. I. Abdallah [2], Sherif M. Afifi [3], Abdelbaset M. Elgamal [4], Abdel Razik H. Farrag [5], Abd El-Nasser G. El-Gendy [6], Mohamed A. Farag [7,8], Abdelsamed I. Elshamy [9,*]

[1] Therapeutic Chemistry Department, Pharmaceutical and Drugs research Institute, National Research Centre, 33 El Bohouth St., Dokki, Giza, 12622, Egypt; nm.ammar@nrc.sci.eg (N.M.A.); ha.el-saud@nrc.sci.eg (H.A.H.)
[2] Pharmacology Department, Medical Research and clinical studies institute, National Research Centre, 12622, Dokki, Giza, Egypt; heba21_5@yahoo.com
[3] Pharmacognosy Department, Faculty of Pharmacy, University of Sadat City, Sadat City 32897, Egypt; shshsh38@hotmail.com
[4] Chemistry of Microbial and Natural Products Department, Pharmaceutical and Drugs research Institute, National Research Centre, 33 El-Bohouth St., Dokki, Giza 12622, Egypt; ; algamalgene@yahoo.com
[5] Department of Pathology, Medical Research and clinical studies institute, National Research Centre, 33 El Bohouth St. Dokki, Giza 12622, Egypt; ar.hussein@nrc.sci.eg
[6] Medicinal and Aromatic Plants Research Department, Pharmaceutical and Drugs research Institute, National Research Centre, Cairo, Egypt; aggundy_5@yahoo.com
[7] Pharmacognosy Department, College of Pharmacy, Cairo University, Kasr el Aini St., Cairo P.B. 11562, Egypt; mfarag73@yahoo.com
[8] School of Forestry and Biotechnology, Zhejiang A&F University, Lin'an, 311300 China
[9] Chemistry of Natural Compounds Department, Pharmaceutical and Drugs Research Institute, National Research Centre, Giza 12622, Egypt

* **Correspondence**: elshamynrc@yahoo.com or ai.el-shamy@nrc.sci.eg;Tel.: +20-100-552-5108 (A.I.E.)

No	Supplementary data	Page
1	Figure S1: Area of BCl2 immnuohistochemical expression in hepatic tissue measured by the image analysis system as area percent. Data are expressed as Mean ± SE (n = 6). Statistical analysis was carried out by one-way ANOVA using Tukey as post-hoc test. [a] Significantly different from Control negative at $P < 0.05$. [b] Significantly different from Control positive at $P < 0.05$.	2
2	Figure S2:: Area of BCl_2 immnuohistochemical expression in renal tissue measured by the image analysis system as area percent. Data are expressed as Mean ± SE (n = 6). Statistical analysis was carried out by one-way ANOVA using Tukey as post-hoc test. [a] Significantly different from Control negative at $P < 0.05$. [b] Significantly different from Control positive at $P < 0.05$.	3
3	Table S1. Relative percentile of silylated primary metabolites in sera from all groups using SPME-GC–MS (n = 6), results are presented as average ± (std. deviation)	4-7
4	Figure S3. The representative chromatograms of serum samples from different animal groups	8

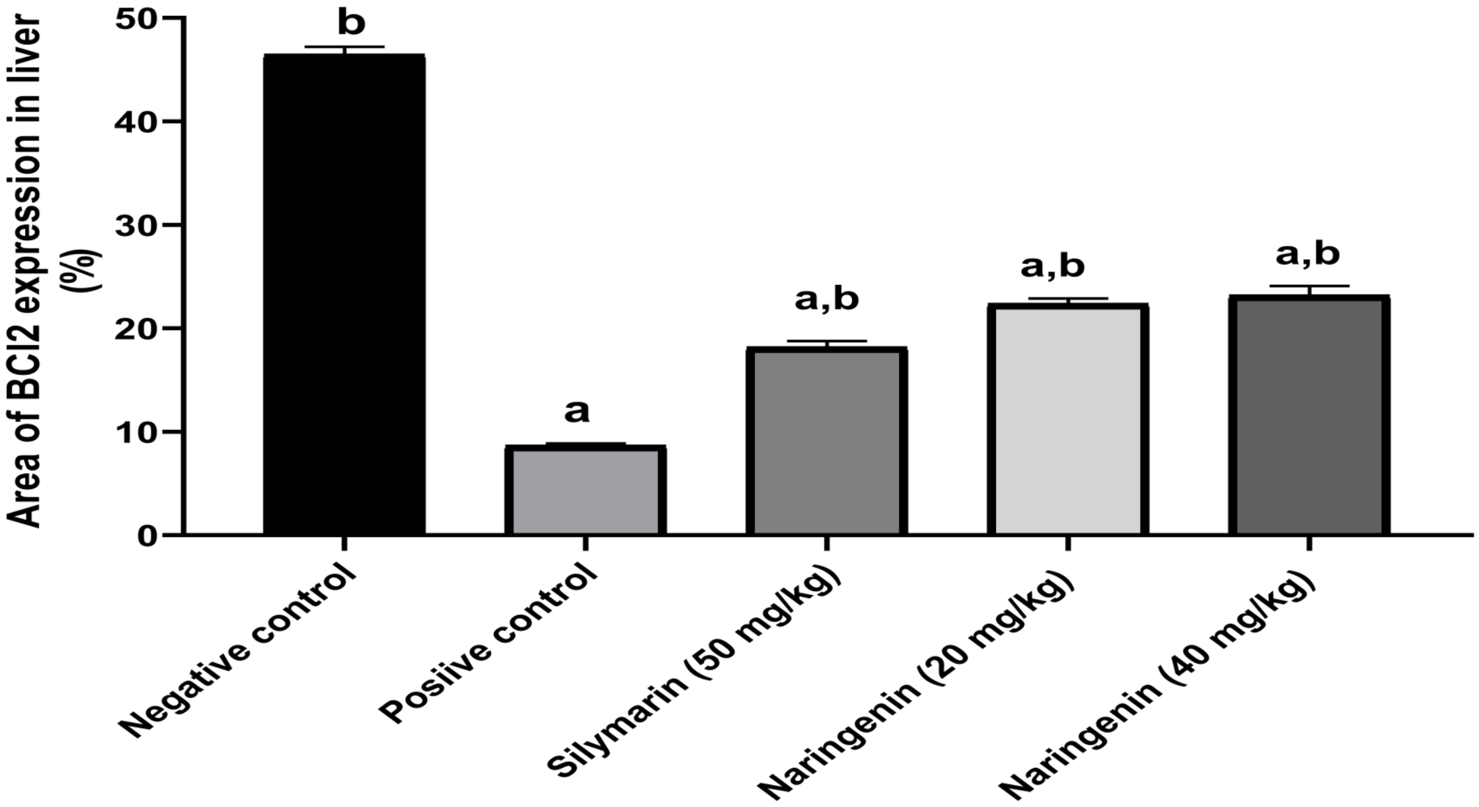

Figure S1: Area of BCl2 immnuohistochemical expression in hepatic tissue measured by the image analysis system as area percent. Data are expressed as Mean ± SE (n = 6). Statistical analysis was carried out by one-way ANOVA using Tukey as post-hoc test. [a] Significantly different from Control negative at $P < 0.05$. [b] Significantly different from Control positive at $P < 0.05$.

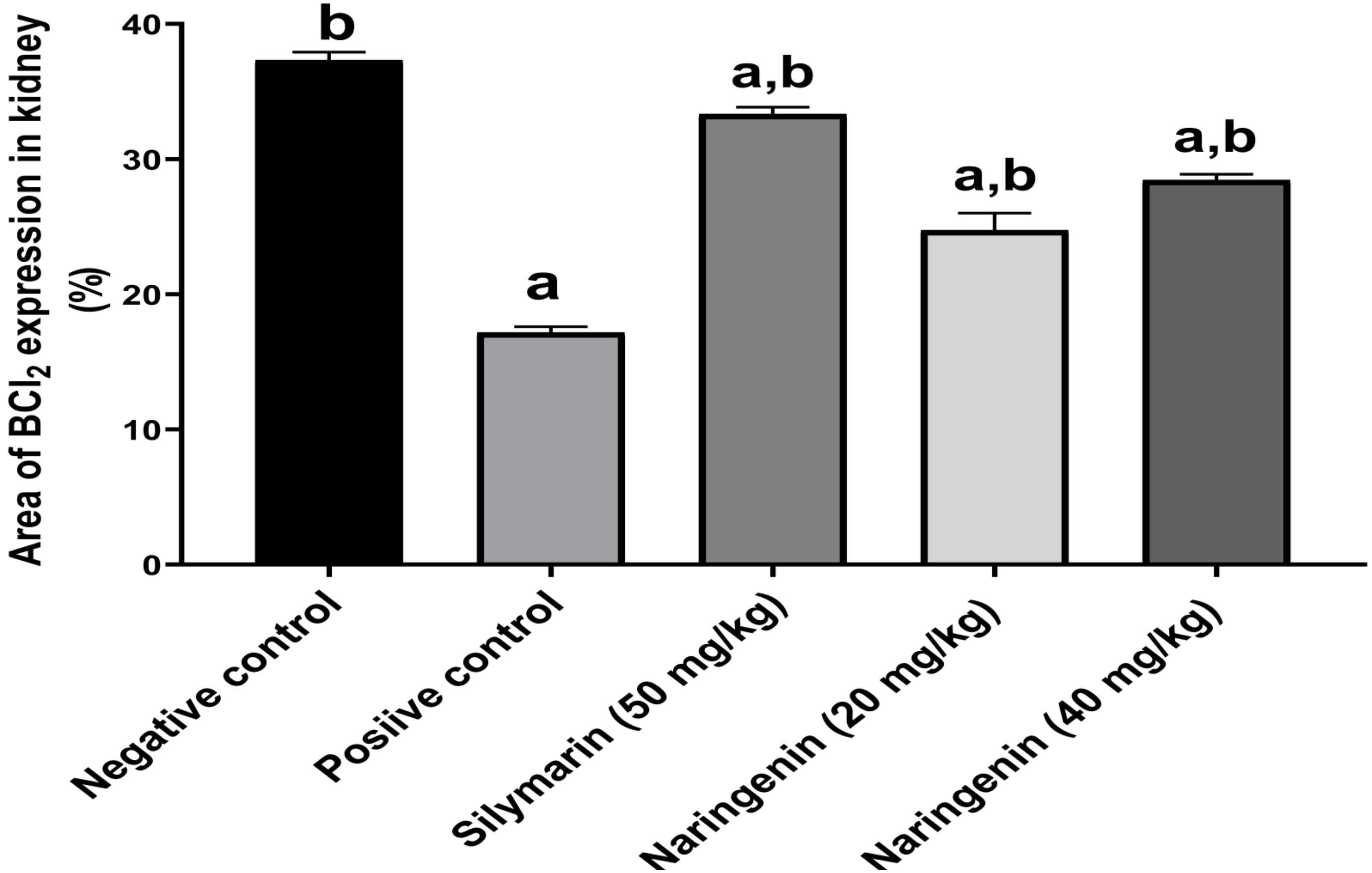

Figure S2:: Area of BCl_2 immnuohistochemical expression in renal tissue measured by the image analysis system as area percent. Data are expressed as Mean ± SE (n = 6). Statistical analysis was carried out by one-way ANOVA using Tukey as post-hoc test. [a] Significantly different from Control negative at $P < 0.05$. [b] Significantly different from Control positive at $P < 0.05$.

Table S1. Relative percentile of silylated primary metabolites in sera from all groups using SPME-GC–MS (n = 6), results are presented as average ± (std. deviation)

No.	Rt(min)	KI	Name	Control	Nar. 20 mg	Nar. 40 mg	CCL_4	Silymarin
	**Amino acids**							
1	5.051	1099	L-Alanine, 2TMS	0.66 ± 0.57	0.45 ± 0.51	0.68 ± 0.88	0.45 ± 0.40	0.43 ± 0.57
2	5.095	1102	L-Alanine, 2TMS isomer	0.81 ± 0.31	0.41 ± 0.32	0.61 ± 0.51	0.45 ± 0.30	0.45 ± 0.35
3	5.452	1122	Glycine, 2TMS	0.01 ± 0.01	0.00 ± 0.01	-------	0.01 ± 0.01	0.01 ± 0.01
4	7.015	1208	L-Valine, 2TMS	0.08 ± 0.08	0.11 ± 0.12	0.11 ± 0.13	0.09 ± 0.11	0.11 ± 0.12
5	8.451	1316	L-Isoleucine, 2TMS	0.04 ± 0.02	0.04 ± 0.04	0.04 ± 0.04	0.06 ± 0.06	0.04 ± 0.05
6	8.586	1321	L-Proline, 2TMS	0.20 ± 0.16	0.06 ± 0.09	0.07 ± 0.10	0.09 ± 0.08	0.07 ± 0.09
7	8.659	1324	Glycine, 2TMS isomer	0.67 ± 0.21	0.44 ± 0.22	0.27 ± 0.14	0.46 ± 0.31	0.55 ± 0.39
8	9.681	1362	Serine, 3TMS	0.39 ± 0.42	0.31 ± 0.29	0.41 ± 0.25	0.64 ± 0.44	0.61 ± 0.55
9	10.084	1377	L-Threonine, 3TMS	0.54 ± 0.15	0.75 ± 0.43	0.72 ± 0.40	0.69 ± 0.48	0.83 ± 0.54
10	12.62	1468	L-Methionine, 2TMS	0.16 ± 0.06	0.06 ± 0.03	0.06 ± 0.03	0.09 ± 0.06	0.06 ± 0.04
11	12.753	1473	Pyroglutamic acid, 2TMS	0.81 ± 0.26	0.40 ± 0.25	0.31 ± 0.23	0.53 ± 0.38	0.54 ± 0.38
12	14.151	1591	L-Ornithine, 3TMS	0.02 ± 0.01	0.01 ± 0.00	0.01 ± 0.01	0.01 ± 0.01	-------
13	14.281	1604	L-Glutamic acid, 3TMS	0.01 ± 0.00	-------	-------	-------	0.00 ± 0.01
14	14.413	1612	Phenylalanine, 2TMS	0.25 ± 0.11	0.13 ± 0.07	0.13 ± 0.08	0.14 ± 0.08	0.14 ± 0.09
15	16.787	1761	L-Glutamine, 3TMS	0.01 ± 0.01	0.01 ± 0.01	0.01 ± 0.02	0.01 ± 0.00	0.01 ± 0.01
16	17.212	1788	L-Ornithine, 4TMS	0.11 ± 0.04	0.12 ± 0.09	0.05 ± 0.03	0.15 ± 0.11	0.13 ± 0.09
17	18.778	1896	L-Lysine, 4TMS	0.36 ± 0.09	0.22 ± 0.15	0.17 ± 0.16	0.19 ± 0.13	0.25 ± 0.16
18	19.139	1921	L-Tyrosine, 3TMS	0.26 ± 0.12	0.07 ± 0.05	0.07 ± 0.04	0.12 ± 0.11	0.10 ± 0.08
19	22.862	2152	L-Tryptophan, 3TMS	0.80 ± 0.23	0.31 ± 0.18	0.39 ± 0.24	0.23 ± 0.21	0.27 ± 0.18
	Total amino acids			6.19	3.90	4.12	4.42	4.61
	**Amino sugars**							
20	22.221	2114	Glucosamine, 6TMS	1.89 ± 1.14	2.57 ± 1.32	4.70 ± 2.13	2.49 ± 1.33	3.30 ± 1.46
	Total amino sugars			1.89	2.57	4.70	2.49	3.30

	**Esters**							
21	16.438	1738	Glycerol-3-phosphate, 4TMS	0.01 ± 0.00	-------	-------	0.01 ± 0.01	-------
	Total esters			0.01	-------	-------	0.01	-------
	**Fatty acids**							
22	9.891	1370	Nonanoic acid, TMS	0.03 ± 0.02	0.02 ± 0.02	0.02 ± 0.02	0.04 ± 0.04	0.03 ± 0.03
23	11.644	1434	8-Methylnonanoic acid, TMS	0.02 ± 0.01	0.02 ± 0.02	0.01 ± 0.02	0.04 ± 0.03	0.02 ± 0.03
24	14.964	1646	Lauric acid, TMS	0.43 ± 0.32	0.44 ± 0.45	0.30 ± 0.32	0.61 ± 0.64	0.57 ± 0.67
25	18.011	1843	Myristic acid, TMS	0.19 ± 0.20	0.23 ± 0.30	0.15 ± 0.21	0.57 ± 0.94	0.34 ± 0.52
26	18.022	1843	Myristic acid, TMS isomer	0.36 ± 0.24	0.37 ± 0.38	0.23 ± 0.28	0.67 ± 0.88	0.48 ± 0.55
27	19.432	1941	Pentadecanoic acid, TMS	0.04 ± 0.03	0.04 ± 0.05	0.03 ± 0.04	0.17 ± 0.29	0.06 ± 0.09
28	20.822	2031	Palmitic Acid, TMS	3.27 ± 0.77	3.13 ± 1.63	2.36 ± 1.25	10.43 ± 17.54	3.22 ± 1.35
29	20.856	2033	Palmitic Acid, TMS isomer	2.63 ± 1.69	2.23 ± 2.38	1.65 ± 1.94	7.73 ± 12.05	2.55 ± 2.22
30	21.433	2067	Methyl linoleate	0.06 ± 0.03	0.04 ± 0.04	0.13 ± 0.12	0.08 ± 0.10	0.07 ± 0.05
31	22.101	2107	Margaric acid, TMS	0.18 ± 0.07	0.17 ± 0.10	0.13 ± 0.08	0.32 ± 0.36	0.16 ± 0.12
32	23.038	2162	Oleic acid, TMS	0.21 ± 0.13	0.13 ± 0.13	0.14 ± 0.12	0.30 ± 0.39	0.30 ± 0.26
33	23.115	2166	Oleic acid, TMS	0.06 ± 0.06	0.06 ± 0.09	0.07 ± 0.10	0.18 ± 0.33	0.09 ± 0.16
34	23.128	2167	Petroselinic acid, TMS	0.14 ± 0.10	0.14 ± 0.16	0.13 ± 0.15	0.35 ± 0.42	0.18 ± 0.23
35	23.392	2183	Stearic acid, TMS	0.76 ± 0.59	0.78 ± 1.01	0.64 ± 0.76	1.95 ± 2.38	0.79 ± 1.06
36	24.89	2271	Arachidonic acid, TMS	0.07 ± 0.02	0.03 ± 0.02	0.04 ± 0.04	0.04 ± 0.05	0.04 ± 0.03
	Total fatty acids			8.44	7.83	6.04	23.47	8.87
	**Inorganic acids**							
37	8.183	1306	Phosphoric acid, 3TMS	0.94 ± 0.37	0.93 ± 0.72	0.49 ± 0.32	0.92 ± 0.53	1.01 ± 0.33
38	8.278	1310	Phosphoric acid, 3TMS isomer	1.00 ± 0.40	0.72 ± 0.49	0.42 ± 0.24	0.69 ± 0.49	0.53 ± 0.38
	Total inorganic acids			1.94	1.65	0.91	1.61	1.54
	**Nitrogenous compounds**							
39	5.131	1105	Hydroxylamine, 3TMS	0.68 ± 0.43	0.17 ± 0.13	0.04 ± 0.03	0.45 ± 0.29	0.27 ± 0.28
40	6.07	1155	2-Piperidinone, TMS	-------	0.01 ± 0.01	0.01 ± 0.01	0.01 ± 0.02	-------

41	7.813	1281	Urea, 2TMS	0.11 ± 0.07	0.08 ± 0.07	0.08 ± 0.11	0.07 ± 0.06	0.07 ± 0.09
42	8.059	1301	Urea, 2TMS isomer	0.71 ± 0.20	0.44 ± 0.30	0.46 ± 0.30	0.47 ± 0.31	0.36 ± 0.23
43	8.116	1303	Urea, 2TMS isomer	2.82 ± 0.39	2.21 ± 1.45	1.44 ± 1.12	2.35 ± 1.52	1.75 ± 1.26
44	13.101	1486	Creatinine, 3TMS	0.01 ± 0.00	-------	0.02 ± 0.03	-------	-------
45	19.011	1913	Palmitonitrile	0.09 ± 0.03	0.07 ± 0.04	0.25 ± 0.17	0.13 ± 0.14	0.12 ± 0.07
46	21.528	2073	9-Octadecenenitrile	1.19 ± 0.15	1.03 ± 0.52	2.15 ± 0.62	1.19 ± 0.58	1.38 ± 0.50
47	21.56	2075	9-Octadecenenitrile isomer	0.37 ± 0.12	0.46 ± 0.15	0.83 ± 0.50	1.10 ± 0.68	0.99 ± 0.47
48	25.552	2309	Octadecanamide, TMS	3.43 ± 0.62	3.59 ± 0.79	3.03 ± 0.82	3.09 ± 1.51	3.88 ± 1.80
49	25.561	2311	Octadecanamide, TMS isomer	4.09 ± 0.52	4.44 ± 1.38	3.78 ± 0.80	3.99 ± 2.36	4.35 ± 1.15
50	25.834	2326	Stearamide, TMS	0.04 ± 0.02	0.03 ± 0.03	0.03 ± 0.02	0.12 ± 0.20	0.04 ± 0.04
51	26.558	2370	Docos-9-enenitrile	0.42 ± 0.22	0.54 ± 0.25	1.11 ± 0.27	0.63 ± 0.31	0.69 ± 0.27
52	29.913	2568	(Z)-Docos-13-enamide, N-TMS	1.83 ± 0.60	1.22 ± 0.68	2.73 ± 0.56	1.56 ± 0.81	2.21 ± 0.87
	Total nitrogenous compounds			15.79	14.29	15.97	15.16	16.11
	**Organic acids**							
53	3.649	1017	Caproic acid, TMS	0.22 ± 0.18	0.02 ± 0.03	0.02 ± 0.03	0.08 ± 0.07	0.07 ± 0.06
54	4.377	1060	Lactic Acid, 2TMS	3.67 ± 3.37	8.77 ± 5.06	10.10 ± 3.02	1.98 ± 3.35	4.65 ± 5.17
55	4.445	1063	Lactic Acid, 2TMS isomer	8.58 ± 2.55	8.96 ± 3.55	6.89 ± 3.11	1.18 ± 6.27	8.05 ± 3.68
56	4.733	1081	Glycolic acid, 2TMS	0.01 ± 0.00	0.02 ± 0.02	0.02 ± 0.02	0.02 ± 0.02	0.02 ± 0.02
57	5.723	1136	Oxalic acid, 2TMS	0.01 ± 0.01	0.01 ± 0.01	0.20 ± 0.49	0.01 ± 0.02	0.00 ± 0.01
58	5.803	1140	Oxalic acid, 2TMS isomer	1.43 ± 1.15	1.17 ± 0.62	1.86 ± 0.78	1.75 ± 1.24	0.76 ± 0.89
59	6.101	1156	3-Hydroxybutyric acid, 2TMS	0.35 ± 0.52	0.03 ± 0.02	0.03 ± 0.02	0.06 ± 0.04	0.07 ± 0.06
60	6.35	1170	Octanoic acid, TMS	0.01 ± 0.01	0.02 ± 0.02	0.01 ± 0.02	2.03 ± 0.05	0.02 ± 0.02
61	7.524	1255	4-Hydroxybutanoic acid, 2TMS	0.01 ± 0.01	0.01 ± 0.01	0.01 ± 0.01	2.01 ± 0.01	0.01 ± 0.01
62	9.027	1338	Succinic acid, 2TMS	0.01 ± 0.01	0.01 ± 0.01	-------	2.02 ± 0.03	0.01 ± 0.02
63	10.615	1397	3-Deoxytetronic acid, 3TMS	0.04 ± 0.03	-------	-------	-------	-------
64	11.036	1412	3-Methylglutaric acid, 2TMS	0.01 ± 0.01	0.01 ± 0.01	-------	2.01 ± 0.01	0.01 ± 0.01
	Total organic acids			14.37	19.03	19.15	13.17	13.69

	**Sterols**							
65	32.901	2744	Cholesterol, TMS	0.26 ± 0.08	0.08 ± 0.04	0.17 ± 0.13	0.13 ± 0.12	0.18 ± 0.10
	Total sterols			0.26	0.08	0.17	0.13	0.18
	**Sugars**							
67	17.731	1823	1-Deoxyglucose, 4TMS	0.84 ± 0.34	0.26 ± 0.13	0.21 ± 0.12	0.27 ± 0.21	0.27 ± 0.12
68	18.236	1858	Glucose, methyloxime, 5TMS	9.05 ± 3.09	13.62 ± 5.33	10.98 ± 4.37	11.39 ± 9.97	12.47 ± 5.68
69	18.266	1860	Glucose, methyloxime, 5TMS	9.15 ± 2.98	9.13 ± 7.69	10.98 ± 4.37	2.80 ± 3.34	8.40 ± 8.24
70	18.475	1875	Galactose, methyloxime, 5TMS	7.05 ± 0.91	6.80 ± 0.92	6.06 ± 0.54	4.52 ± 2.66	6.02 ± 1.08
	Total sugars			26.09	29.81	28.23	18.98	27.16
	**Sugar alcohols**							
71	8.045	1300	Glycerol, 3TMS	3.03 ± 0.37	2.48 ± 1.55	2.18 ± 1.15	2.04 ± 1.60	2.08 ± 1.43
72	15.298	1666	D-(+)-Arabitol, 5TMS	7.01 ± 1.21	3.11 ± 3.70	4.03 ± 2.60	3.94 ± 3.73	6.25 ± 2.66
73	15.324	1668	D-(+)-Arabitol, 5TMS isomer	7.01 ± 1.21	7.72 ± 1.56	5.58 ± 0.88	7.23 ± 3.94	7.52 ± 1.77
74	16.217	1724	Ribitol, 5TMS	0.01 ± 0.00	0.01 ± 0.00	-	0.01 ± 0.01	0.01 ± 0.00
	Total sugar alcohols			17.05	13.31	11.79	13.22	15.86
	**Sugar lactones**							
75	19.251	1929	Glucuronic acid γ-lactone,methyloxime, 3TMS	0.18 ± 0.09	0.07 ± 0.06	0.11 ± 0.08	0.12 ± 0.17	0.08 ± 0.09
76	19.599	1953	Glucuronic acid γ-lactone,methyloxime, 3TMS isomer	0.06 ± 0.04	0.02 ± 0.02	0.09 ± 0.10	0.02 ± 0.02	0.02 ± 0.02
	Total sugar lactones			0.24	0.09	0.21	0.13	0.10
	**Unknowns**							
77	8.883	1332	Unkonwn 1	0.71 ± 0.09	0.64 ± 0.29	0.60 ± 0.26	0.56 ± 0.33	0.53 ± 0.28
78	18.664	1889	Unknown 2	0.17 ± 0.08	0.06 ± 0.07	0.03 ± 0.03	0.25 ± 0.38	0.17 ± 0.17
79	18.669	1889	Unknown 3	0.14 ± 0.04	0.07 ± 0.05	0.03 ± 0.03	0.15 ± 0.13	0.14 ± 0.07
80	18.931	1907	Unknown 4	0.33 ± 0.24	0.36 ± 0.42	0.37 ± 0.44	0.79 ± 1.31	0.48 ± 0.73

81	20.177	1992	Unknown 5	0.62 ± 0.23	0.24 ± 0.12	0.16 ± 0.08	0.23 ± 0.17	0.25 ± 0.17
82	24.361	2240	Unknown 6	5.76 ± 0.82	6.06 ± 2.68	7.52 ± 2.34	5.23 ± 2.96	7.01 ± 1.66
	Total unknowns			7.73	7.43	8.71	7.21	8.58

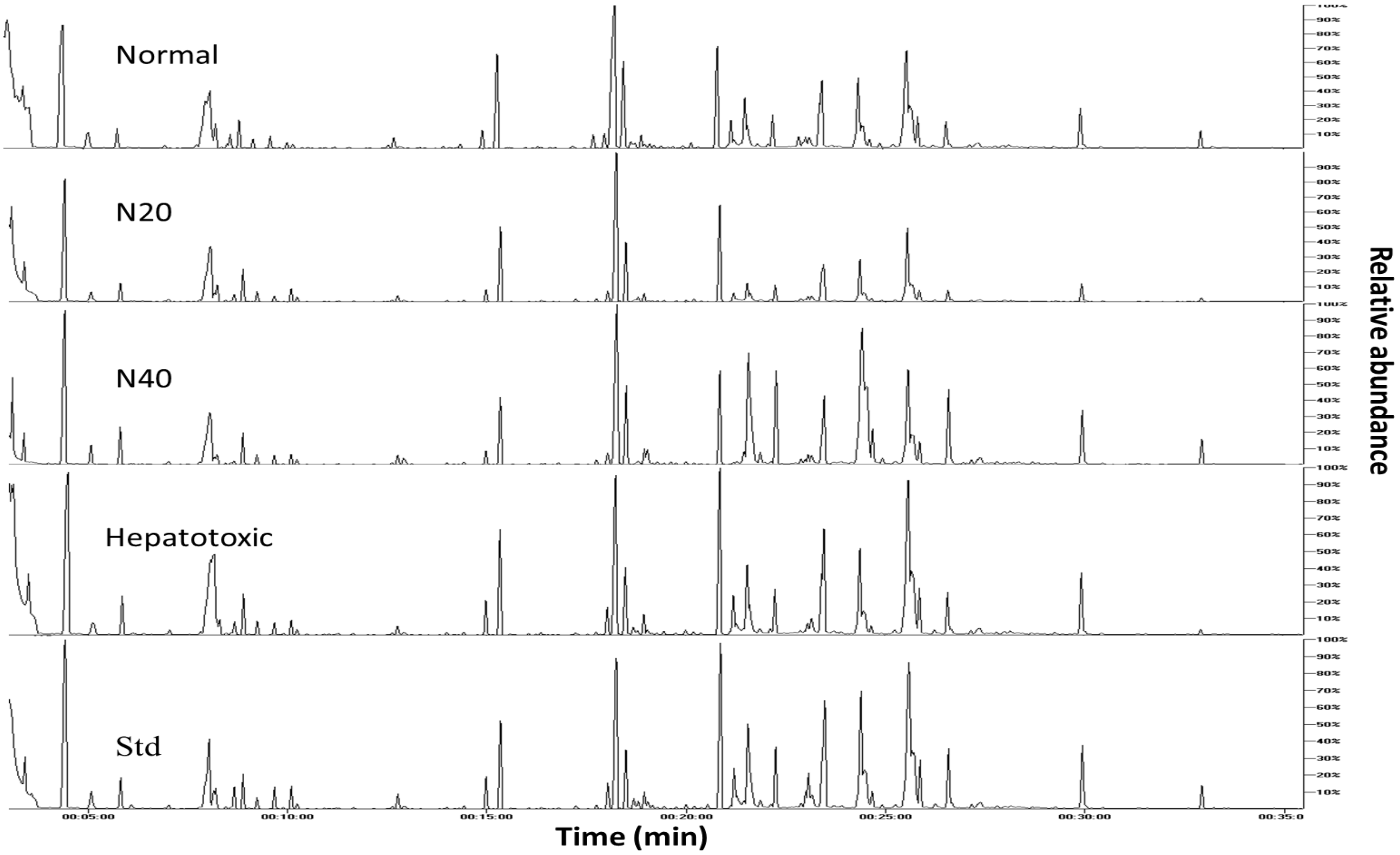

Figure S3. The representative chromatograms of serum samples from different animal groups. • N20: naringenin at 20 mg/kg, N40: naringenin at 40 mg/kg, Std: standard drug (silymarin).
